# ALternatives To prophylactic Antibiotics for the treatment of Recurrent urinary tract infection in women (ALTAR): study protocol for a multicentre, pragmatic, patient-randomised, non-inferiority trial

**DOI:** 10.1186/s13063-018-2998-4

**Published:** 2018-11-09

**Authors:** Rebecca Forbes, Ased Ali, Alaa Abouhajar, Catherine Brennand, Heather Brown, Sonya Carnell, Thomas Chadwick, Ian Eardley, Jan Lecouturier, Helen Mossop, Ian Pearce, Robert Pickard, Nikesh Thiruchelvam, Katherine Walton, Jennifer Wilkinson, Chris Harding

**Affiliations:** 1 Newcastle Clinical Trials Unit, Newcastle University, 1–4 Claremont Terrace, Newcastle upon Tyne, NE2 4AE UK; 2 Department of Urology, Freeman Hospital, Freeman Road, Newcastle upon Tyne, NE7 7DN UK; 3 Institute of Cellular Medicine, William Leech Building, The Medical School, Newcastle upon Tyne, NE2 4HH, UK; 4 Addenbrooke’s Hospital, Cambridge, CB2 0QQ UK; 5 St James’s University Hospital, Leeds, LS9 7TF UK; 6 Manchester Royal Infirmary, Manchester, M13 9WL UK; 7 Institute of Health and Society, Newcastle University, Newcastle upon Tyne, NE2 4AX UK; 8 Pinderfields Hospital, Wakefield, WF1 4EE UK; 9 Department of Microbiology, Freeman Hospital, Newcastle upon Tyne, NE7 7DN UK

**Keywords:** Antibiotic resistance, Antibiotic prophylaxis, Economic evaluation, Qualitative research, Randomised controlled trial, RCT, Urinary tract infection, UTI

## Abstract

**Background:**

At least half of all adult women will experience infective cystitis (urinary tract infection: UTI) at least once in their life and many suffer from repeated episodes. Recurrent urinary tract infection (rUTI) in adult women is usually treated with long-term, low-dose antibiotics and current national and international guidelines recommend this as the ‘gold standard’ preventative treatment. Although they are reasonably effective, long-term antibiotics can result in bacteria becoming resistant not only to the prescribed antibiotic but to other antimicrobial agents. The problem of antimicrobial resistance is recognised as a global threat and the recent drive for antibiotic stewardship has emphasised the need for careful consideration prior to prescribing antibiotics. This has led clinicians and patients alike to explore potential non-antibiotic options for recurrent UTI prevention.

**Design /methods:**

This is a multicentre, pragmatic, patient-randomised, non-inferiority trial comparing a non-antibiotic preventative treatment for rUTI in women, methenamine hippurate, against the current standard of daily low-dose antibiotics. Women who require preventative treatment for rUTI are the target population. This group is comprised of those with a diagnosis of rUTI, defined as three episodes in 1 year or two episodes in 6 months, and those with a single severe infection requiring hospitalisation. Participants will be recruited from secondary care urology / urogynaecology departments in the UK following referral with rUTI. Participants will be followed up during a 12-month period of treatment and in the subsequent 6 months following completion of the prophylactic medication. Outcomes will be assessed from patient recorded symptoms, quality of life questionnaires and microbiological examination of urine and perineal swabs. The primary outcome is the incidence of symptomatic antibiotic-treated UTI self-reported by participants during the 12-month period of preventative treatment. Health economic outcomes will also be assessed to define the cost-effectiveness of both treatments. A qualitative study will be conducted in the first 8 months of the trial to explore with participants/non-participants’ and recruiting clinicians’ views on trial processes and identify potential barriers to recruitment, reasons for participating and non-participation and for dropping out of the study.

**Discussion:**

The study was commissioned and funded by the National Institute for Health Research (NIHR) and approved under the Medicines and Healthcare products Regulatory Agency (MHRA) notification scheme as a ‘Type A’ study.

**Trial registration:**

International Standard Randomised Controlled Trial Number (ISRCTN), registry number: ISRCTN70219762. Registered on 31 May 2016.

**Electronic supplementary material:**

The online version of this article (10.1186/s13063-018-2998-4) contains supplementary material, which is available to authorized users.

## Background

Recurrent urinary tract infection (rUTI) in adult women is one of the most common infections in humans. Bacteria from the vagina or faecal matter inoculate the periurethral area, then the bladder, causing symptoms of cystitis. The lifetime risk of a urinary tract infection (UTI) is around 40% in adult women and the incidence peaks in the third and ninth decades. The annual incidence of a single UTI is 3% [1] with up to 44% of these women experiencing recurrence within 1 year [2]. This equates to an affected population of over 300,000 women in 1 year in the UK [3].

The prophylactic treatment of rUTI with extended courses of low-dose antimicrobial therapy is standard practice and a meta-analysis from the Cochrane Collaboration shows that this reduces recurrence rate by up to 80% [4]. However, antibiotics are the main driving force in the development of antibiotic resistance and can lead to resistance not only of the causative microorganisms but also of the commensal flora [5]. Prudent antibiotic prescribing (stewardship) is one of the cornerstones of UK and international strategies to prevent antimicrobial resistance [6, 7] and recent guidelines have identified that ‘repeated/prolonged treatment with antibiotics’ is contributory to the development of this serious health problem [8]. Alternative, non-antibiotic treatments for the prevention of rUTI have the potential to improve public health by minimising the development of antimicrobial resistance in bowel reservoirs and well-designed trials are needed to demonstrate their effectiveness.

## The current body of evidence and the contribution of this study

Prophylactic antibiotics constitute the current standard of care for the preventative treatment of rUTIs and are recommended for this use by both UK and European guidelines [8, 9] .The largest meta-analysis examining the efficacy of prophylactic antibiotics reveals an 85% reduction in symptomatic UTI over placebo (RR 0.15, 95% CI 0.08 to 0.28) [4]. This meta-analysis included 19 studies with data from 1120 women. The authors concluded that continuous antibiotic prophylaxis for 6–12 months reduced the rate of UTI during prophylaxis when compared to placebo. There were, however, more adverse events (AEs) in the antibiotic group and these included vaginal and oral candidiasis and gastrointestinal symptoms. The observation that following treatment the rate of symptomatic UTI returned to similar levels in both women taking prophylactic antibiotics and those receiving placebo comes from only two studies. This does, however, suggest that the benefits of antibiotics are not sustained following cessation of treatment.

Methenamine hippurate has been the subject of a meta-analysis from the Cochrane Collaboration [10] and this included 13 studies comprising data from 2043 patients. The reduction in UTIs was of the order of 76% (RR 0.24, 95% CI 0.07 to 0.89). The authors did however comment that the quality of the included studies was mixed and that pooled estimates for the major outcome measures were not interpretable because of underlying heterogeneity. Nevertheless, they were able to assert that methenamine hippurate may be effective for preventing UTI in patients without renal tract abnormalities, particularly when used for short-term prophylaxis. The rate of AEs was low, but poorly described. The need for large, well-conducted clinical trials to clarify the effectiveness of methenamine hippurate in the setting of prevention of UTIs was highlighted.

Although continuous antibiotic treatment has been shown to prevent rUTIs [4] previous randomised trials have demonstrated an associated threefold increase in the incidence of antimicrobial resistance compared with placebo [11]. Several studies have confirmed the emergence of resistant organisms in the faecal reservoir and urine of women who take prolonged antibiotics [11, 12]. The resistance pattern observed was not confined to the prescribed antibiotic but to a range of other antibiotic agents commonly used to treat symptomatic UTI [1]. Furthermore the detection of resistant microorganisms can occur after just a few weeks of prophylactic antibiotic therapy [11]. The use of effective non-antibiotic UTI prevention strategies will reduce risk to patients of emergence of resistant organisms and subsequent difficult-to-treat clinical infection with these bacteria. The incidence of antimicrobial multi-resistance within post-menopausal women suffering from rUTI is around 25% and was shown to rise to more than 80% following prolonged antibiotics [5].

The advisability of using non-antibiotic preventative treatments for rUTI has been highlighted by recent UK, European and US guidelines to reduce the ‘collateral damage’ of antibiotic use by minimising risk of resistance development [6]. Policy-makers in the UK and Internationally have included antibiotic avoidance and prudent antibiotic prescribing as key components of action plans and strategies to reduce antimicrobial resistance [13, 14]. A well-designed research study providing robust evidence of at least equivalent effectiveness of non-antibiotic treatment is needed to further inform guideline-writers and policy-makers and allow recommendation of alternative treatments avoiding prolonged antibiotic use.

In addition to the problem of antimicrobial resistance within an individual’s faecal reservoir there is a wider public health concern regarding the continued emergence of healthcare-associated infections (HAI) initially focussed on methicillin resistant *Staphylococcus aureus* (MRSA) and *Clostridium difficile* infection (CDI) and more recently other types of multi-resistant organisms including those producing extended-spectrum beta lactamases (ESBL). The predominant uropathogen, *Escherichia coli (E. coli)*, is the subject of a recent article identifying the overuse of non-prescription antibiotics in Asia as a potential causative factor for the development of a new mechanism of ESBL antibiotic resistance detected in the UK [15]. Limiting the use of broad-spectrum antibiotics is a key measure in addressing this problem, and has been the driver for recent UK guideline updates [8]. The development of antimicrobial stewardship programmes which encourage prudent antibiotic prescribing has already been shown to reduce antibiotic use and consequently the incidence of HAI which until recently was increasing [16, 17]. Avoidance of antibiotic administration, where possible, is believed to be the single most important factor leading to the observed decline in HAI in Scotland [17]. A positive result from our study will further encourage avoidance of antibiotic prescription by providing high-level evidence of efficacious non-antibiotic alternatives reducing the chance of emergence of multi-resistant UTIs.

Our study will address the question of whether the use of the urinary antiseptic methenamine hippurate (a non-antibiotic preventative treatment) results in an equivalent reduction in rate of symptomatic UTIs compared to prophylactic antibiotic therapy in women suffering from rUTI. In addition, we will assess the longer-term effectiveness of these two treatments by following up all participants for 6 months after treatment. We will also evaluate both treatments in terms of their cost-effectiveness and effect on overall quality of life (QoL). Furthermore, secondary outcomes will assess whether use of this alternative preventative treatment reduces the development of antimicrobial resistance within uropathogens that is associated with antibiotic use including the main pathogen *E. coli*. Using health economic analysis, we aim to value this benefit to women suffering rUTI and to the wider NHS.

## Why this research is needed now

A recent meta-analysis reviewed the evidence for non-antibiotic treatments as prophylaxis against rUTI but the results were disappointing, mainly due to paucity of evidence [18]. One of the conclusions in this report was that ‘Although sometimes statistically significant, pooled findings for the other (non-antibiotic) interventions should be considered tentative until corroborated by more research’. It would appear that one of the barriers to clinicians recommending non-antibiotic alternatives for the treatment of rUTI is the lack of currently available clinical evidence. The campaign for antibiotic stewardship and more prudent prescribing of antibiotic agents can only be strengthened by further work exploring effectiveness of non-antibiotic alternatives. A further conclusion from this meta-analysis was that ‘Large head-to-head trials should be performed to optimally inform clinical decision-making’.

The most recent guideline published on the subject of UTI is the 2012 Scottish Intercollegiate Guideline Network (SIGN) guideline 88 [8] which forcibly illustrates why our proposed study is essential and needed promptly. The literature review carried out prior to formulation of this document identified ‘considerable evidence of practice variation’ and variation in ‘initiation of antibiotic treatment’ for UTI. In addition, one of the constant themes in this report is the need to avoid ‘unnecessary antibiotic prescribing’ which is associated with ‘clinical adverse events including *Clostridium difficile* infection (CDI) or methicillin-resistant *Staphylococcus aureus* (MRSA) infection, and the development of antibiotic-resistant (*E. coli*) UTIs’. The UK antimicrobial resistance strategy and action plan [6] states that ‘the increasing prevalence of antimicrobial resistant micro-organisms is causing international concern’ and identifies that ‘the emergence of resistance represents adaptive selection by micro-organisms which is an inevitable result of therapeutic use of antimicrobial agents’. This document reflects an urgent need for prudent antibiotic use as one of three key elements of the strategy to control antibiotic resistance. The ALTAR study aims to provide high-level contemporary evidence of the relative effectiveness of a non-antibiotic UTI prevention treatment compared to the standard treatment of prolonged, low-dose antibiotics within a UK population of women with rUTI in a routine NHS care setting.

## Design/methods

The ALTAR trial is a robust, pragmatically designed trial to evaluate the clinical benefit and cost-effectiveness of the best candidate alternative treatment for the prevention of rUTI, the urinary antiseptic methenamine hippurate compared to the standard treatment of low-dose prophylactic antimicrobial therapy. Outside of the trial medication, both groups will receive usual care and any breakthrough UTI will be treated by discrete courses of antibiotic treatment.

The null hypothesis being tested is that the non-antibiotic treatment (methenamine hippurate) is inferior to the standard treatment of an extended course of prophylactic antibiotic for the prevention of rUTI in women and less cost-effective to the NHS. The alternative hypothesis is that methenamine hippurate is as good at preventing UTI and is as cost-effective as antibiotic prophylaxis. We have chosen to investigate the possible non-inferiority of methenamine hippurate in order to clarify an alternative treatment choice to antibiotics in treating rUTIs, which may prevent increases in antibiotic-resistant strains of infection.

Estimates of prevalence, effectiveness and harms from Cochrane reviews have informed the power calculation conservatively based on what we, guided by a patient panel, considered to be a minimum threshold difference that would drive patient and clinician acceptability together with change of practice prompted by inclusion of trial results in future meta-analyses and guidance for management of rUTI in the NHS and internationally.

**Fig. 1:**
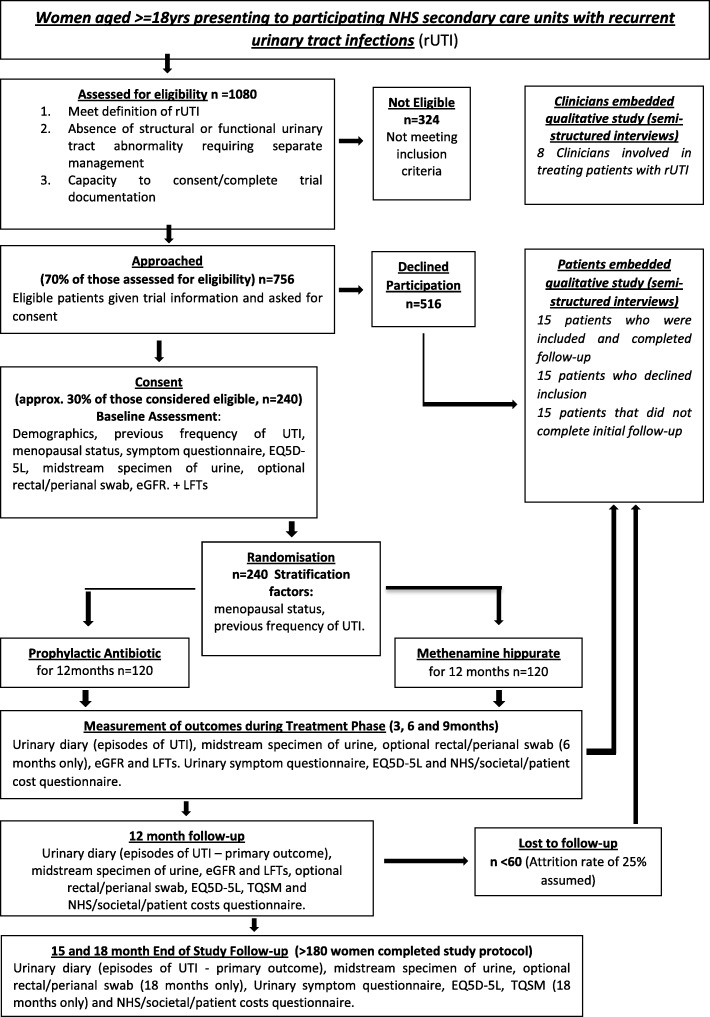
Trial flow chart

The anticipated trial flow for participants is shown in Fig. 1  and the trial schedule of procedures is shown in Table 1.

**Table 1:** Schedule of procedures

Procedures	Screening	Baseline	Treatment phase						Follow up	
			3 months	6 months	9 months	12 months	At time of UTI	Monthly checks	15 months	18 months
Informed consent		X								
Demographics	X	X^a^								
Medical history		X								
Physical examination		X								
eGFR and LFTs (a sample for DNA will be taken at one of these time points)	X	X^a^	X	X	X	X			X	X
MSU (local laboratory)		X	X	X	X	X	X		X	X
MSU (central laboratory)		X	X	X	X	X	X		X	X
Perineal swab		X		X		X				X
Concomitant medications	X	X^a^								
Eligibility assessment	X									
Randomisation		X								
Dispensing of trial drugs		X	X	X	X					
Compliance			X	X	X	X	X	X	X	X
UTI record							X			
UTI questionnaire			X	X	X	X			X	X
EQ5D-5 L		X	X	X	X	X	X		X	X
Health Resource Use Questionnaire		X	X	X	X	X				X
TQSM						X				X
Adverse event assessments			X	X	X	X	X		X	X
CRF completion	X	X	X	X	X	X	X	X	X	X
Qualitative interviews	X^b^					X^c^				

*CRF* Case Report Form, *DNA* deoxyribonucleic acid, *eGFR* estimated glomerular filtration rate, *EQ5D L* EuroQoL 5 Dimension Questionnaire, *LFT* liver function tests, *MSU* mid-stream urine, *TQSM* Treatment Questionnaire on Satisfaction with Medication, *UTI* urinary tract infection

^a^Screening data values may be used for baseline if taken within 14 days from date of randomisation. ^b^15 patients who declined to participate in main study but consented to interview study. ^c^15 patients who do not complete the treatment and 15 patients who stay in the study up to 6 months post randomisation will be interviewed. Time points will vary

## Objectives

### Primary objective

The primary objective is to:

- Determine the relative clinical effectiveness and cost-effectiveness for the NHS of two types of licensed preventative treatments for women with recurrent uncomplicated urinary tract infection (rUTI) over a 12-month treatment period

### Secondary objectives

The secondary objectives are to determine:

- The relative impact on the incidence of symptomatic antibiotic-treated UTI self-reported by patients during the 6-month follow-up period after completion of 12 months of allocated treatment
- The total number of days spent on urinary specific antibiotics (prophylactic or treatment) during the 12-month treatment period and 6 months of follow-up
- If there is any longitudinal ecological change in terms of phenotype and genotype of bacteria and their resistance patterns in isolates from individual participant’s (1) urine and (2) faecal reservoir during the 12-month treatment period and in the 6 months following completion of treatment
- The number of microbiologically proven UTIs during the 12-month treatment and 6-month follow-up periods
- The incidence of asymptomatic bacteriuria (ABU) during the study period
- The incidence rate of hospitalisation due to UTIs during the study period
- Overall patients’ satisfaction with antibiotic vs antiseptic treatment
- Patients’ and clinicians’ views regarding trial processes and participation via an embedded qualitative study
- The incremental cost per Quality-adjusted Life Year (QALY) gained at 18-month periods based on responses to the EuroQol 5 Dimension Questionnaire (EQ5D-5 L)
- The incremental costs to the NHS, personal social services measured at the end of the 18-month study period
- The relative health economic efficiency over the patient’s lifetime using a modelling exercise

### Primary outcome measures

The primary outcome measures for the ALTAR trial are:

1. Incidence of symptomatic antibiotic-treated UTI self-reported by participants and verified where necessary from medical records during the 12-month period of preventative treatment
2. Incremental cost per QALY gained during the 12-month treatment period. Incremental costs to the NHS, personal social services, and the patient at 12 months

Occurrence of a symptomatic UTI will be captured by:

- Participants completing a UTI record when experiencing a UTI
- Face-to-face appointments and Case Report Form (CRF) completion at 3, 6, 9 and 12 months
- Participant questionnaires completed at 3, 6, 9 and 12 months
- Monthly contact with the local site trial team
- End-of-trial review and primary care record review

The incremental costs to the NHS, personal social services and the patient at 12 months will be captured by:

- Treatment costs of drugs and healthcare services from standard NHS sources such as the *British National Formulary* (*BNF*)
- Health Resource Use Questionnaires at 3, 6, 9 and 12 months

### Secondary outcome measures

1. The number of symptomatic, antibiotic-treated UTIs self-reported by participants in the 6-month follow-up period after completing the allocated preventative therapy
2. Total antibiotic use during the study period, reported by patients and verified where necessary from medical records
3. Phenotype and genotype of *Escherichia coli* (*E. coli*) isolated from urine and perineal swabs sent by participants directly to the central reference laboratory
4. The number of microbiologically confirmed UTIs occurring during both the 12 months of treatment and the subsequent 6 months of follow-up. A positive culture will be classified according to standard Public Health England (PHE) definitions; the laboratory report of two isolates at ≥10^5^ cfu/mL or a single isolate at ≥10^4^ cfu/mL
5. Presence of ABU identified by urine culture performed at patient visits for study follow-up. ABU is defined as the presence of bacteria in the urine in the absence of symptoms suggestive of UTI. For the purposes of this study, a positive culture is defined in line with the routine PHE definitions above
6. The incidence rate of hospitalisation due to UTIs during the treatment and follow-up phases of the study
7. Overall satisfaction with treatment measured by Treatment Questionnaire on Satisfaction with Medication (TQSM)[19] administered at both the end of treatment (12 months) and then again at the end of follow-up (18 months)
8. Qualitative analysis of patients’ and clinicians’ views regarding trial processes and participation
9. QALYs based on responses to the EQ5D-5 L at 3, 6, 9, 12, 15 and 18 months and after a UTI episode
10. Treatment costs for drug and healthcare services from a standard NHS source such as the *BNF* and published tariffs from NHS reference costs
11. Health Resource Use Questionnaire at 3, 6, 9, 12, 15 and 18 months. Incremental cost per QALY gained during the total 18-month trial period. Incremental costs to the NHS, personal social services, and the patient at 18 months
12. Costs and QALYs will be combined in a cost-utility analysis for both a ‘within’ trial analysis and modelled over the patient’s lifetime using previously developed methods and data from other relevant randomised controlled trials (RCTs) that collected patient costs

These will be captured by:

- Participants completing a UTI record when experiencing a UTI
- At face-to-face appointments and CRF completion at 3, 6, 9, 12, 15 and 18 months
- In participant questionnaires completed at 3, 6, 9 and 12 months
- Monthly contact with the local site trial team
- Results from a mid-stream specimen of urine (MSU) and perineal swabs
- End-of-trial review and primary care record review

### Study setting

Large, secondary care urology centres with a consistent clinical assessment pathway for women with rUTI will be selected as sites for this multicentre clinical trial. Centres will be sufficiently resourced and have a proven track record of delivering clinical research with established links to their respective Clinical Research Networks (CRN). The principal investigator (PI) or delegated individual will be responsible for coordinating participant recruitment by screening women with rUTI who are routinely referred from primary care to these centres. We initially plan to open four sites and we will consider opening further sites if the rate of recruitment is slower than anticipated.

### Patient identification

We will aim to ensure that all adult women referred to each site with rUTI are aware of the study prior to their clinic appointment and those eligible can consider whether they wish to participate prior to assessment. Each research site lead will publicise the study within their own departments and referral catchment areas and ensure that colleagues in allied specialities, such as urogynaecology and nephrology, who may receive referrals of women with rUTI, are aware of the study and are willing to identify potential participants. We will use established CRN links to ensure that referring GPs are aware of the study; can identify potential eligible participants and direct referrals accordingly, we will register general practices or other secondary sites as participant identification centres (PIC) if needed. All sites will have an established clinical research track record and effective infrastructure in place for patient recruitment.

In order for the results from the ALTAR study to be generalisable across the wider NHS, the demographic mix of patients recruited to the study must reflect that of patients currently being referred to urologists. Recurrent UTI is generally defined as three episodes of infection within a 12-month period [8] and the patient group most affected by rUTI is adult women, making up over 80% of all people presenting with UTI [15]; this will constitute the majority of our target population. We have expanded the inclusion criteria to other groups that would also be considered for antibiotic prophylaxis including women who have had two episodes of UTI in the preceding 6 months and patients who have had one episode of serious UTI resulting in hospitalisation in the preceding 12 months. Furthermore, patients who are being treated by their general practitioner (GP) in primary care will also be identified by liaison of the lead clinician in each site with primary care leads within Local Clinical Research Networks (LCRNs).

ALTAR study sites will consist of large UK urology/urogynaecology centres, with the majority of referrals coming from primary care through the standard NHS ‘Choose and Book’ pathway. These centres have well-defined existing clinical pathways in place for the investigation of such patients which initially focusses on the exclusion of underlying structural or functional abnormalities of the urinary tract. This is usually done by renal tract ultrasound scan (USS) and an endoscopic examination of the bladder under local anaesthesia (flexible cystoscopy). A recent local audit in Newcastle (unpublished data, *n* = 200) has revealed that contributory structural or functional abnormalities are detected in less than 10% of patients. Therefore, we estimate that approximately 90% of patients referred with rUTI to these centres will be eligible to be approached for inclusion to the ALTAR study. We will compare ratios of screened to randomised patients throughout the trial which will enable us to estimate recruitment rates and ensure that targets are met.

PICs will be considered as a means to maximise recruitment at each site. Participants will be identified by the PIC and information about the study will be provided. Any participants interested in the study will be referred to the main site for possible recruitment into the study through the usual recruitment procedures.

Recruitment will be carried out by research staff in each of the centres and will involve a clear explanation of the trial including the background, study protocol and aims.

### Study design and duration

This is a multicentre, pragmatic patient-randomised, non-inferiority trial comparing two treatments for the prevention of rUTI in women during a 12-month period of treatment and in the 6 months following treatment completion. The standard treatment is once-daily prophylactic antibiotic, using either trimethoprim 100 mg, nitrofurantoin 50 or 100 mg or cefalexin 250 mg once daily for 12 months which are the recommended drugs licensed for this purpose. The choice of antibiotic will be decided by considering previous bacterial sensitivities, safety, and patient or clinician preference. The alternative (experimental) treatment is a 1-g twice daily dose of the orally administered urinary antiseptic methenamine hippurate for 12 months. Participants in both arms would continue to receive treatment courses of antibiotic for UTI as needed.

### Target population

The target population for the ALTAR trial are women (aged > 18 years) with recurrent UTI, for whom prophylactic antibiotics would be considered as a therapeutic option, e.g. at least three episodes of symptomatic antibiotic-treated urinary infection in the previous 12 months, two episodes of UTI in the last 6 months or a single occurrence of severe UTI requiring hospital admission in the preceding 1 year.

### Inclusion criteria

The inclusion criteria are as follows:

- Women aged 18 years and over
- Women with rUTI who, in consultation with a clinician, have decided that prophylaxis is an appropriate option (to include women who have suffered at least three episodes of symptomatic UTI within the preceding 12 months or two episodes in the last 6 months or a single severe infection requiring hospitalisation)
- Able to take a once daily oral dose of at least one of nitrofurantoin, or trimethoprim or cefalexin
- Able to take methenamine hippurate
- Women who agree to take part in the trial but who are already taking methenamine or antibiotic prophylaxis will be consented for participation and will stop their preventative therapy for a 3-month washout period. They will then be reassessed and, if still eligible, undergo baseline assessment and randomisation
- Able to give informed consent for participation in the trial
- Able and willing to adhere to an 18-month study period

### Exclusion criteria

The exclusion criteria are as follows:

- Women who are unable to take methenamine hippurate, e.g. known allergy to methenamine hippurate, severe hepatic impairment (Child-Pugh class C, score of 10 or more, gout, estimated glomerular filtration rate (eGFR) < 10 mL/min, *Proteus* spp. as a consistent proven causative organism for rUTIs
- Women who are unable to take nitrofurantoin and trimethoprim and cefalexin
- Women with correctable urinary tract abnormalities that are considered to be contributory to the occurrence of rUTI
- Presence of symptomatic UTI – this will be treated and symptoms resolved prior to randomisation
- Pregnancy or intended pregnancy in next 12 months
- Women who are breast-feeding
- Women already taking methenamine or antibiotic prophylaxis and declining a 3-month washout period

## Screening, recruitment and consent procedures

### Screening clinical records and face to face

Clinical staff at each site will identify eligible participants through direct contact or by searches of electronic records held in each trust. They will then give or send potentially eligible patients brief study information. Interested potential participants can then agree to be approached by research staff and provided with further study information. Trial invitation information will include brief details of the need and purpose of the study and eligibility criteria. It will emphasise the pragmatic nature of the study and give a realistic indication of the burden to participants. All patients given trial information will be recorded in the screening logs at each site. All subjects who agree to consider participation will be seen by local research staff or the trial coordinator at the respective site to go through the consent and randomisation procedure. A CRF will be initiated and baseline data collected.

A screening log will be kept by local site research staff to document details of subjects invited to participate in the study and reasons for non-participation. Non-identifying patient details and reasons for non-participation will be uploaded to the study electronic Case Report Form (eCRF) for subsequent analysis. The log will also ensure that potential participants who are ineligible or decline participation are approached only once. Participants who do not respond to written information about the study may be contacted a second time to ensure that they have received the information and been given the opportunity to participate.

### Consent

Participants will be consented for randomisation, trial participation, consent regarding telephone interviews, storage of blood, urine and swab for future research and whether they agree to be approached for further research studies in this area.

All participants will undergo a process of informed consent undertaken by appropriately trained staff from the main trial sites. The consent process will include provision of balanced written information concerning the need and overall benefit of the trial followed up by discussion with a local trial coordinator.

In relation to the qualitative interviews, recruiting staff will also explain why it is important to understand why people do and do not participate and how an interview study can help to improve the way that trials are conducted. Participants who are willing to be approached will be provided with a separate information sheet about the interview study.

Following receipt of information about the study, participants will be given at least 24 h and up to as much time as they need to decide whether or not they would like to participate. Written informed consent will always be obtained prior to randomisation. The participant will specifically consent to their GP being informed of their participation in the study. The right to refuse to participate without giving reasons will be respected.

During study set up we will consider requests for trial participant literature including the information sheet and consent form to be translated into other languages. Ability of the participant or their carer to complete the primary outcome questionnaires in English will be required for trial participation. If local NHS circumstances permit, sign interpreters will be arranged for all visits with patients who require them for deaf patients wishing to take part in the study. Interpreters will be used where necessary to explain the consent form and information sheet; great priority will be placed on finding the most direct means of communication. If local research staff are in any doubt with regards to patient understanding of crucial aspects of the trial or ability to collect the outcome measures in English, then consent for randomisation will not be sought.

Participants will be given the option of consenting to storage of blood, urine and a perianal swab for future research. They will also be asked if they would be willing for the inclusion of data collected for this study in future research. Any further research would be subject to separate review by an ethics committee.

### Randomisation

Randomisation will be administered centrally by the Newcastle Clinical Trials Unit (CTU) secure web-based system. Permuted random blocks of variable length will be used to allocate participants 1:1 to the antibiotic and antiseptic groups. An individual not otherwise involved with the study will produce the final randomisation schedule. Stratification by two variables; prior frequency of UTI (less than four episodes per year or more than four episodes per year), and menopausal status of participants (pre-menopausal or menopausal/post-menopausal) will be performed prior to randomisation to ensure balanced allocation within these factors.

Following randomisation an appointment will be arranged, facilitated by trial staff, with the prescribing clinician to commence allocated treatment and ensure continued supply for the 12-month treatment period usually through hospital prescription or via the participant’s GP. The antibiotic selected for use as prophylaxis will be chosen by the patient and clinician with regard to individual participant characteristics, local guidance and standardised trial information with preferred agents being: nitrofurantoin first, trimethoprim second, cefalexin third.

### Blinding

There is no participant blinding in this study. The members of the local research team that will carry out the follow-up process will not be blinded to the allocated treatment for each participant. We will, however, try to ensure that central trial staff will be unaware of allocated group in data reported during the trial where possible.

### Intervention

Apart from random allocation to either option, all participants will receive usual care including use of on-demand discrete treatment antibiotic courses for UTI. We have formulated a recruitment plan to progressively build to a target of 240 participants over an 18-month recruitment window.

### Methenamine hippurate

For those women randomised to receive methenamine hippurate a twice daily dose of 1 g to be taken 12 h apart will be prescribed for 12 months (as recommended in the *BNF*). An eGFR of less than 10 mL/min will be an exclusion criterion for the study. Other exclusion criteria will be patients with gout which is a contra-indication to treatment with methenamine and those with liver dysfunction as determined by pre-study serum liver function tests (LFT) (analysis of blood sample). Patients randomised to receive methenamine hippurate or antibiotic prophylaxis will have blood samples taken at 3, 6, 9 and 12 months to monitor kidney and liver function (eGFR and LFT). If there are any abnormalities in these tests during the period of treatment then a further sample will be taken at 18 months to ensure that these have resolved. If clinically indicated, blood tests may be more frequent. If there are specific and intolerable side-effects, such as nausea, gastrointestinal disturbance, itching or skin rashes, then participants will be given the opportunity to discontinue treatment and be offered an alternative treatment which may include prophylactic antibiotic. This information will be recorded and the participant will continue on the study. If a participant in the methenamine group develops symptoms and signs suggestive of breakthrough UTI then they will seek treatment in their usual way, predominantly by contacting their GP and starting a discrete treatment course of antibiotics. They will be requested to submit a urine sample via their healthcare practitioner, and to send a urine sample to the central reference laboratory before starting treatment, and will be instructed to continue taking methenamine during this antibiotic treatment course. Details of all treatment antibiotic courses will be recorded including the agent used and the number of days that participants actually took the prescribed antibiotic. The rate of UTI will be defined firstly as a simple incident rate and secondly as the incident density rate as described above and annualised for the purpose of standardisation.

### Antibiotic prophylaxis

For those women randomised to receive antibiotic, once-daily, low-dose, prophylactic antimicrobial therapy will be prescribed for 12 months. The agent to be used will be active against common urinary pathogens and selected by the responsible clinician depending on patient characteristics such as previous use, allergy, renal function, liver function, prior urine cultures and local guidance. Available evidence suggests the use of nitrofurantoin 50 mg or 100 mg, trimethoprim 100 mg or cefalexin 250 mg, in that order of preference. Renal function will be determined by eGFR at baseline, and if this is less than 45 mL/min/1.73m^2^ nitrofurantoin will not be used. Patients randomised to receive antibiotic prophylaxis will have blood samples taken at 3, 6, 9 and 12 months to monitor kidney and liver function (eGFR and LFT). If there are any abnormalities in these tests during the period of treatment then a further sample will be taken at 18 months to ensure that these have resolved. If clinically indicated then blood tests may be more frequent. Participants will be asked to take the once-daily antibiotic prophylaxis as a single dose at bedtime. If there are specific and intolerable adverse effects, such as nausea with nitrofurantoin, or candidiasis with cefalexin, then switching to an alternative agent would be advised in consultation with the relevant clinician with the reasons for the change recorded. The aim will be to maintain participants on antibiotic prophylaxis using any one of the three agents for as long as possible during the 12-month treatment period within tolerance and safety constraints. Participants intolerant of prophylactic antibiotic despite trying alternative agents will have the opportunity to discontinue the medication and be offered an alternative treatment which may include methenamine hippurate. This information will be recorded and the participant will continue on study. If a participant in the antibiotic prophylaxis group develops symptoms and signs suggestive of breakthrough UTI then they will seek treatment in their usual way mostly by contacting their GP and starting a discrete treatment course of antibiotics following submission of a mid-stream specimen of urine for routine culture and also to the central laboratory. In this scenario they will be instructed to stop the prophylactic antibiotic while they are taking a treatment course and restart it again the day following the last dose they take of the treatment course. Clinicians and participants will be advised to use a different agent for treatment than the one they are taking for prophylaxis. Details of all treatment antibiotic courses will be recorded including the agent used and the number of days the participants actually took the prescribed antibiotic. The rate of UTI will be defined firstly as a simple incidence rate and secondly as the incident density rate; the number of UTIs suffered during the observation period minus days spent taking treatment courses of antibiotics active against urinary tract organisms. This number will be annualised for the purposes of standardisation.

### Standard of care for participants

This trial is pragmatic in design and, apart from random allocation of treatment option and participant completion of diaries and questionnaires; participant care will follow standard pathways in participating secondary care NHS sites. Both prophylactic antibiotic and methenamine hippurate are licensed and approved for routine NHS use. We will ensure that all participants have access as desired to the use of other measures to reduce the risk of UTI such as adequate fluid intake, avoidance of constipation, and, for post-menopausal women, vaginally administered oestrogen supplements. We will also ensure that all participants are informed regarding the possible benefit of other alternative options including cranberry extract. Participants in both trial groups may receive discrete courses of antibiotics as decided by the responsible clinician for symptomatic UTI. Use of all these adjunctive treatments will be recorded on CRFs.

### Concomitant medication

It is the responsibility of the prescribing clinician to check for interactions between trial drugs and other medications.

### Baseline

The following procedures will be undertaken at the baseline visit, prior to randomisation, but after the participant has given informed consent:

- Demographic review/document eligibility including UTI details (stratify UTI frequency for randomisation)
- Pre/post menopause (stratify for randomisation)
- Document adjunctive treatments, e.g. cranberry/ oestrogens/ D-mannose/probiotics
- eGFR and LFTs, plus optional sample for storage and DNA analysis
- Health Resource Use Questionnaire
- EQ5D-5 L
- Mid-stream specimen of urine (MSU) for microscopy and culture plus MSU for central laboratory
- Optional perineal swab for culture for the presence of *E.coli* (central laboratory only)

### Randomisation

Participants will be randomised onto the study as close to the time of consent as possible. Participants who are undertaking the 3-month washout will consent to the study prior to undertaking the washout but will not be randomised or complete the other baseline measures until this has been completed. Their continued consent and eligibility will be confirmed at this point.

### Post randomisation

Post randomisation there will be a discussion of the trial documentation with the participant and the trial medication will be prescribed (either methenamine hippurate or antibiotic prophylaxis). Site staff will also go through the instructions for what to do if the participant experiences a UTI and how to complete the UTI record and take and send the associated samples.

### Once-monthly telephone calls

A member of the trial team will contact the participant once monthly (when they are not attending a visit in person) to check compliance, any concerns of the participant and tolerance of their allocated investigational medicinal product (IMP).

### Face-to-face appointments at 3, 6, 9, 12, 15 and 18 months

Participants will attend a face-to-face appointment every 3 months. At this appointment the following will be completed:

- UTI diary review
- Completion of CRF by trial staff
- MSU for microscopy and culture (plus MSU for central laboratory)
- Optional perineal swab (central laboratory only at 6, 12 and 18 months)
- eGFR and LFTs blood tests
- Prescription for trial medication (3, 6 and 9 months only)

### At the time of UTI

At the time of the participant experiencing a UTI, they will complete and return:

- Participant UTI record
- EQ5D 5 L
- MSU for culture and microscopy (plus MSU for central laboratory)
- Trial staff to complete report alert after telephone call from participant

### Questionnaires sent directly to participants

A number of questionnaires will be sent directly to participants from the central team at the Newcastle Clinical Trials Unit. These are as follows:

- UTI Symptom Questionnaire
- EQ5D 5 L
- Health Resource Use Questionnaire
- TQSM

### Microbiological methods

MSU samples will be sent to the central reference laboratory in universal containers pre-loaded with boric acid at a concentration of 18 g/L (International Scientific Supplies Ltd., Bradford, UK). Microscopy and semi-quantitative culture of mid-stream specimens of urine will be carried out in accordance with the UK Standards for Microbiology Investigations [26]. The presence of up to two isolates at ≥1 × 10^5^ cfu/mL or one isolate at ≥1 × 10^4^ cfu/mL will be reported, while bacterial counts of ≤10^3^ cfu/mL and mixed cultures of three isolates or more will be regarded as not significant. Presumptive identification will be confirmed by matrix-assisted laser desorption ionisation-time of flight mass spectrometry, MALDI-TOF (Bruker microflex, Coventry, UK). Disc diffusion susceptibility testing against a panel of antimicrobial agents will be carried out using Mueller-Hinton agar (Oxoid Limited, Basingstoke, UK) in accordance with the methods outlined by The European Committee on Antimicrobial Susceptibility Testing (EUCAST) [27]. For *E. coli* isolates, susceptibility testing will be carried out in triplicate. Perianal swabs will be inoculated onto ChromID® CPS® Elite media (bioMérieux, Basingstoke, UK) and examined for the presence of *E. coli* after overnight incubation in room air at 37 °C. As above, antimicrobial susceptibility testing will be carried out in triplicate for *E. coli* strains using EUCAST disc diffusion methodology [27].

### Subject withdrawal

Participants have the right to withdraw from the trial at any time without having to give a reason. Investigator sites should try to ascertain the reason for withdrawal and document this reason within the CRF and participant’s medical notes.

The investigator may discontinue a participant from the trial at any time if the investigator considers it necessary for any reason including:

- Pregnancy
- Participant withdrawal of consent
- Investigator’s discretion that it is in the best interest of the participant to withdraw
- An adverse event (AE) that renders the participant unable to continue in the trial
- Termination of the clinical trial by the sponsor

Participants who withdraw from the trial will not be replaced. There are three withdrawal options:

1. Withdrawing completely (i.e. withdrawal from allocated treatment and provision of follow-up data, including follow-up through patient healthcare records)
2. Withdrawing from the allocated treatment (moving to the alternative treatment arm) in the trial but allowing continued full follow up (including questionnaires) and review by research team of healthcare records
3. Withdrawing from the allocated treatment in the trial and the active follow-up but allowing the research team to follow-up through healthcare records

A proportion of participants who discontinue participation in the study will be invited to take part in the qualitative interviews as it is important to understand why some participants withdraw from the trial.

### Definition of the end of study

The definition of the end of the trial will be the last participant’s last follow-up visit at 18 months post randomisation. An end-of-trial declaration will be submitted to the REC and MHRA.

### Source of bias

Selection bias will be minimised by including all adult female patients with recurrent uncomplicated UTI as eligible participants. We have deliberately set few exclusion criteria to enable the findings of this study to be generalisable. Both treatments are licensed for this condition, exhibit a low side-effect profile. Target population and sample size and have little interaction with other common medications, which limits absolute contra-indications to either therapy. We will stratify randomisation on the basis of number of UTIs (more than four episodes per year vs four or more episodes per year) and menopausal state (pre-menopausal vs menopausal/post-menopausal) of the participants to ensure equivalent proportions of these groups at differential risk in both arms.

Eligible patients and their responsible clinicians will need to be sufficiently uncertain of the optimum treatment for rUTI to allow randomisation. The ‘Background and Rationale’ section (sections ‘1 and 2’) of this document sets out the existing evidence for both treatments and describes Level-1 evidence to support the use of both prophylactic antibiotics and methenamine hippurate. Similar reductions in the frequency of episodes of UTI are reported for both treatments and clinicians should, therefore, have equipoise based on these data. This should ensure that any selection bias in terms of characteristics of rUTI sufferers put forward and willing to be randomised compared with those who are eligible but not willing to participate is minimised. We will keep an anonymised screening log at each centre listing demographic and clinical characteristics and reasons for declining randomisation (if offered) and compare this group with those entering and those completing the trial. Secondly the characteristics of participants who switch treatment arm during the 12-month treatment period may differ from those completing the allocated strategy. We will address this by comparison of demographic data and QoL scores between these groups measured at baseline prior to randomisation and following treatment.

### Data handling and record keeping

Data will be collected using CRFs, participant-completed questionnaires, UTI diaries and information retrieved from medical notes. Data will be entered remotely at each site into the secure validated clinical data management system Elsevier’s MACRO by the local investigator or another member of the site research team with delegated responsibility for this activity. A site delegation log of the study site personnel and their delegated study activities will be kept in the Investigator Site File (ISF) throughout the duration of the study. CRFs, participant-completed questionnaires and UTI diaries which are entered into eCRFs in the MACRO database at a later date will be classed as source documentation. Results of urine and perineal swab analysis will be uploaded securely into the MACRO database by the database manager from reports produced by the central laboratory. Data will be handled, computerised and stored in accordance with the Data Protection Act 1998 and the General Data Protection Regulation (GDPR) from 25 May 2018. Under the trial participant consent, identifiable data will be stored in a separate, password-protected database within Newcastle Clinical Trials Unit (NCTU), with access limited to those members of the trial team responsible for the preparation and sending of follow-up questionnaires and logging their return. This database will also be used to maintain a record of each participant’s preferred method of communication with the trial team, and to ensure that trial correspondence is sent to each participant using their preferred mode of delivery. The quality and retention of study data will be the responsibility of NCTU. All study data will be retained in accordance with the latest Directive on Good Clinical Practice (GCP) and local policy.

Questionnaires returned by post to the trial management office in Newcastle will be entered by the trial administrative team at NCTU. The NCTU trial ,management team in collaboration with the database manager will work closely with local site research teams to ensure that the data are as complete and accurate as possible. The NCTU trial management team will be responsible for chasing missing data with sites. Two reminders will be sent to participants to prompt return of questionnaires. Extensive range and consistency checks are done to enhance the quality of the data.

### Access to data and data security

Caldicott approval for use, transfer and storage of participant identifiable information will be obtained at each site.

All research data will be kept in accordance with Newcastle University’s information security policy (http://www.ncl.ac.uk/itservice/policies/). Newcastle University maintains a series of regular backups and off-site mirror servers to ensure continuity and disaster recovery.

Elsevier’s MACRO database is an electronic data capture system which complies with the requirements of regulatory bodies and maintains an audit trail of any changes to the data. All data stored in MACRO benefit from Elsevier’s hosting service in collaboration with Rackspace which features redundancy and backup measures in case of disaster. Users have password-limited access to the MACRO database, which restrict access to their own particular role and site.

Data collected during the course of the research will be kept strictly confidential and accessed only by members of the trial team. Participant’s details will be stored on a secure database under the guidelines of the Data Protection Act 1998 and the General Data Protection Regulation (GDPR) from 25 May 2018. Participants will be allocated a unique trial number at randomisation which will be used on all study-related forms and questionnaires throughout the duration of the study. This will also allow anonymised versions of the secure database to be available to the trial team and subsequently processed for archiving. Personal data will not be kept for longer than is necessary for the purpose for which it has been acquired.

### Discontinuation rules

Data will be analysed at the end of the study; there are no planned interim analyses. An independent Data Monitoring Committee (DMC) will be convened to undertake independent review. The purpose of this committee will be to monitor efficacy and safety endpoints and will operate according to a written terms of reference linked to the DAMOCLES Charter. Only the DMC will have access to full un-blinded study data, if requested, prior to completion of the trial. All analyses will follow a carefully documented Statistical Analysis Plan (SAP). The DMC will be asked to review and comment on this plan prior to analysis. A single main analysis will be performed at the end of the trial when all follow-up has been completed. The DMC will meet initially to agree terms of reference and other procedures. The final trial report will contain full detail of the analytical methodology. The DMC will meet at least three times, at the start, middle and completion of the study. At the first meeting, the Committee will agree on its charter of operation, and discuss and advise on the inclusion of an interim analysis and possible adoption of a formal stopping rule for efficacy or safety.

### Assessment of study adherence

Some participants or their clinicians will seek to change their allocated group at some point during trial participation either due to lack of efficacy or adverse effects for either treatment. Trial literature will emphasise the need to adhere to the allocated strategy during the 12-month trial period if possible and will record any deviation. Multiple switching between prophylactic antibiotic agents will be allowed. If participants do stop their allocated treatment within the 12-month treatment period or if they re-commence prophylaxis during the subsequent 6-month observation period this will be recorded and the participant will continue on study unless they withdraw consent.

### Sample size calculation

The clinical trial has a planned recruitment target of 240 patients, 120 in each of the treatment arms. If there is an actual difference of 0.6 episodes (in favour of treatment with antibiotics), then two groups of 87 patients are required to be 90% sure that the lower limit of a one-sided 95% confidence interval (or equivalently a 90% two-sided confidence interval) will be above the non-inferiority limit of one UTI episode assuming a standard deviation of 0.9 episodes per year. Total sample size assuming two groups and an attrition rate of 25% = 232, rounded up to 240.

We have discussed extensively the relative merits of non-inferiority against superiority comparison and believe the key issue is that an orally administered urinary antiseptic would be acceptable to the patient group provided that their effectiveness for UTI prevention is no worse than antibiotic prophylaxis and that the burden of adverse effects is similar or better. There is also the key added potential benefit of reduced rates of resistant organisms and subsequent collateral harm to the individual and the community. The sample size calculation is based on the following assumptions:

- Semi-structured interviews with a patient panel of 12 women identified that any reduction in UTI episodes even by one per year would be deemed worthwhile. Therefore, we have set the minimum clinically important difference between the treatment arms of one UTI per 12 months as our non-inferiority margin
- The two existing meta-analyses of studies examining prophylactic antibiotics [6] and methenamine hippurate [7] have quoted mean relative risk of UTI vs placebo of 0.15 and 0.24, respectively. Using these values and data from a local audit (unpublished, *n* = 200) suggesting that the average number of UTI episodes per year in this patient group is 6.5, we have estimated that the difference in number of episodes per year between prophylactic antibiotics and methenamine hippurate to be 0.6 episodes (in favour of antibiotics)
- The standard deviation of episodes of UTI per year is taken from the placebo groups in the studies included in the Cochrane meta-analyses [4, 10] and has been conservatively estimated at 0.9 episodes per year
- The type-1 error rate for a two group comparison is set at 5%, thus the calculation of a one-sided 95% confidence interval (or a two-sided 90% confidence interval)
- The attrition rate of participants in this study has been conservatively estimated at 25%

### Statistical analysis

The main analysis will comprise a comparison of patients randomised to antiseptic with patients randomised to antibiotic (‘intention to treat’).The primary clinical outcome is the occurrence of symptomatic UTI during the 12-month period of treatment. Our hypothesis is that treatment with antiseptic is not inferior to treatment with antibiotic. When considering an inferiority limit the variable that patients most readily relate to is the number of episodes experienced during treatment. The inferiority limit adopted for this study will be one episode per year. A 90% confidence interval for the difference between groups (antibiotic minus antiseptic) will be calculated using a resampling (bootstrap) procedure. Provided that the lower 90% confidence limit is greater than the pre-specified non-inferiority limit (here − 1 for this definition of difference as a higher (worse) value in the antiseptic group would result in a negative difference), we will infer that treatment with antiseptic is not inferior to treatment with antibiotic.

A secondary analysis of the primary outcome will involve the modelling of the number of episodes of UTI using a negative binomial regression model with differences between centre included as a random effect and a binary indicator of previous annual frequency of UTI at baseline (more than four episodes vs four or less episodes) and menopausal status (pre-menopausal vs menopausal/post-menopausal) will be included as fixed effects. This will yield an estimate of the incidence rate ratio. A binary indicator of at least one patient-reported or clinician-recorded symptom of UTI will be analysed using the same approach but with a binomial error structure. The same methods will be used to analyse the relative frequency of episodes of UTI during the 6-month post-treatment period as a secondary outcome.

Analysis of the secondary outcomes will follow a broadly similar strategy although non-inferiority will not be assessed as this is only relevant for the pre-specified primary outcome. Incidence or occurrence type outcomes will be analysed in a manner analogous to that previously described for the primary outcome. Patient satisfaction will be compared between arms using an analysis of variance/covariance approach adjusting for stratification variables and other predefined baseline covariates. Health-related QoL will be analysed as part of the health economics analysis.

We will also undertake a per-protocol analysis. The primary analysis will be repeated but on the subset of patients who have been treated in accordance with the treatment protocol for the arm to which they were randomised. Patients who switch treatments will still be analysed within the group to which they were randomised but only if that switching has been undertaken in accordance with the specified protocol.

A full SAP will be produced and finalised prior to data lock and analysis commencing.

### Qualitative substudy

In the embedded qualitative study which we propose to conduct in the early phase of recruitment, we will carry out in-depth telephone interviews with up to 15 patients in each of three groups (those who agree to participate, those who decline and, if available, those who drop out of the study before the end of the follow-up period). Also, we will conduct telephone interviews with up to eight clinicians recruiting to the trial. This will provide information regarding both patients’ willingness to be randomised and clinicians’ views on treatment randomisation. A descriptive report with proposed action will be prepared and sent to the Trial Steering Committee (TSC) for approval this will include rate and reasons of declining randomisation and participant attrition.

### Qualitative analysis

Topic guides for both patient and clinician telephone interviews will be developed with the input of the study team and Patient and Public Involvement (PPI) group. Interviews will be digitally recorded with the permission of the interviewee and transcribed verbatim. NVivo will be used as a tool to manage and code the transcript data. Data will be analysed drawing upon the constant comparative method. Issues identified that impact on recruitment and are resolvable, such as lack of clarity in the patient study information or consent process, will be addressed immediately. We plan the headline results to be available to inform change in study procedures at an early stage of the recruitment phase.

### Health economics analysis

A ‘within-trial’ and model-based economic evaluation will be conducted. These analyses will take the form of a cost-utility analysis. The within-trial analysis will take the perspective of the NHS and personal and social services, but will also take a wider perspective by including costs by the participants and their families. The model-based analysis will take the perspective of the NHS and personal and social services.

### Within-trial analysis

For each trial participant the use of health and social care services will be recorded. The use of services for the initial treatments (medications), including time in hospital, will be collected on the CRF. Also collected on the CRF will be the use of secondary care services such as duration of any hospital stay, number of outpatient visits, use of tests, and any change in medications. Use of primary care services, such as GP visits, will be collected via questionnaire at baseline, 3, 6, 9, 12 and 18 months. Information of further patient costs will be sourced from other relevant RCTs that collected patient costs due to the burden on respondents from collecting this type of data.

Costs for healthcare services will be obtained from standard sources such as NHS reference Healthcare Resource Group (HRG) tariffs, the *British National Formulary*25 (*BNF*) for medications, and Unit Costs of Health and Social Care [20] for primary care usage. Further data will come from the study centres themselves such as the cost of consumables and other equipment used for treatment. The price-year adopted for the base-case analysis will be the year when the final analysis is conducted. For each participant measures of use of resources will be combined with unit costs to provide a cost for that participant.

The relative changes in health-related QoL resultin from reductions in recurrent UTIs, together with any harms associated with each of the treatment strategies and with subsequent treatments for UTIs, will be captured by the EQ5D-5 L. Tariffs will be used to provide values so that the EQ5D-5 L data can be used for decision-making [25]. Health State Utilities from the EQ5D-5 L will be used to estimate QALYs for each participant using the area-under-the-curve approach.

Data on costs and QALYs will be used to estimate the mean cost and QALYs for each intervention group. The cost and QALY data will then be used to estimate incremental costs and QALYs and incremental costs per QALY. These data will be presented as point estimates and bootstrapping techniques will be used to estimate the statistical imprecision surrounding them. The results of this stochastic analysis will be presented as cost and QALY plots and as cost-effectiveness acceptability curves [21].

### Model-based analysis

Drawing upon existing modelling expertise in the Health Economics Group at Newcastle University, an economic model describing recurrent UTIs will be developed. The model will be constructed following guidelines for best practice in economic modelling [22].

The use of services both for the treatment and management for recurrent UTIs will be modelled and the costs of these events will be based upon the estimates for these events derived from within the trial. The trial-based data will be the main source of data for the economic model but it will be supplemented by focussed searches of the literature and health economic databases (e.g. the Centre for the Evaluation of Value and Risk in Health (CEVR) Cost Effectiveness Analysis (CEA) Registry; NHS Economic Evaluation Database).

Discounting will be applied to costs and outcomes at the UK-recommended rate of 3.5% [23]. Further data required for the model relate to the transition and other probabilities of events occurring over the lifetime of patients. These probabilities include the risk of recurrence as well as probabilities of receiving different types of intervention should recurrence occur.

The model will be used to produce estimates of costs and QALYs (from the EQ5D-5 L). Cost-effectiveness will be reported as incremental cost per QALY gained (at both 12 months and over the patient’s lifetime). The model will be probabilistic and distributions will be attached to all parameters, the shape and type of distribution will depend upon the data available and recommendations for good practice in modelling [24]. The results will also be presented as point estimates of costs, effects, incremental costs, QALYS and measures cost-utility. They will also be presented as plots of costs and QALYs derived from the probabilistic analysis and cost-effectiveness acceptability curves. Deterministic sensitivity analyses will be combined with the probabilistic analysis to explore other forms of uncertainty.

### Trial monitoring

Monitoring of study conduct and data collected will be performed by a combination of central review and site monitoring visits to ensure that the study is conducted in accordance with GCP. Study site monitoring will be undertaken by members of the Trial Management Group (TMG). The main areas of focus will include consent, serious adverse events (SAEs) and essential documents in study. Site monitoring will include:

- All original consent forms will be reviewed as part of the study file; confirmation of the presence of a copy in the patient hospital notes may be requested for 10% participants
- All original consent forms will be compared against the study participant identification list
- All reported SAEs will be verified against clinical records (Source Data

Verification (SDV))

- The presence of essential documents in the ISF and study files will be checked
- Verification of primary endpoint data and eligibility data for 10% of participants entered in the study may be requested

Central monitoring will include:

- All applications for study authorisations and submissions of progress/safety reports will be reviewed for accuracy and completeness, prior to submission
- All documentation essential for study initiation will be reviewed prior to site authorisation
- Statistical monitoring for outlier sites and unusual data patterns

All monitoring findings will be reported and followed up with the appropriate personnel in a timely manner.

Adverse events (AEs) occurring during trial participation will be recorded and reported in line with GCP guidelines. The expected rate of AEs is low for both treatment arms. All non-serious adverse reactions (ARs) will be recorded in the study database for the duration of the trial. Any SAEs or serious adverse reactions (SARs) will be recorded throughout the duration of the trial in the study database and on the specific trial SAE form.

The trial may be subject to audit by representatives of the sponsor (Newcastle upon Tyne Hospitals NHS Foundation Trust) or inspection by the MHRA or HTA. Each investigator site will permit trial-related monitoring, audits and regulatory inspection including access to all essential and source data relating to the trial.

### Trial reporting

Final data cleaning and preparation of tables for analysis, and writing of Background and Methodology sections of the report will commence at month 30 but the results will not be communicated to the PMG and TSC by the trial statistician until after the end of follow-up phase at month 48. These data will be available to the DMC on request or when indicated by the trial statistician.

During this period the health economics team will prepare the structure of the cost-effectiveness analyses using dummy and actual data as it becomes available. Final analysis together with preparation of the introduction and methodology sections of the report and associated peer-reviewed publication will then be performed during months 47–54 with finalisation of the report by end of month 55.

### Dissemination and outputs

The results of the study will be presented at topic-specific national/international conferences and be published in a general medical, infectious diseases or urology themed peer-reviewed journal. The trial will provide high-level evidence to use in new or updates of existing systematic reviews such as those published by Cochrane.

The most significant anticipated outcome from this study will be demonstration of the efficacy of a non-antibiotic treatment for the prevention of recurrent UTIs, methenamine hippurate. If our hypothesis holds true the study will represent a significant step forward in the treatment of recurrent urinary infection, with high-level evidence for the use of a treatment strategy that avoids prolonged antibiotic use which is directly in line with the UK Government’s strategy to combat antimicrobial resistance. It is likely that national and international media will pick up the story and inform the wider public of the results and their significance. We will also engage with relevant NHS managers and other trust representatives to facilitate prompt changes in local practice and promote this alternative preventative treatment for recurrent UTIs. The results will be publicised on both hospital websites and discussed at departmental meetings. The results will be disseminated to members of professional groups, such as BAUS and EAU, through updates and presentations.

Participants will be provided with a lay summary of results. They will also have access to a copy of journal articles through the trial website. Members of our PPI focus groups will review results and they will be involved in writing lay summaries of results for dissemination to relevant patient groups such as the Cystitis and Overactive Bladder Foundation (COB) and the Bladder and Bowel Foundation. We will utilise the COB expertise on how best to deliver these results to the other participants and patient-specific groups. These will be in accessible formats in keeping with equality legislation.

### SPIRIT

**Fig. 2:**
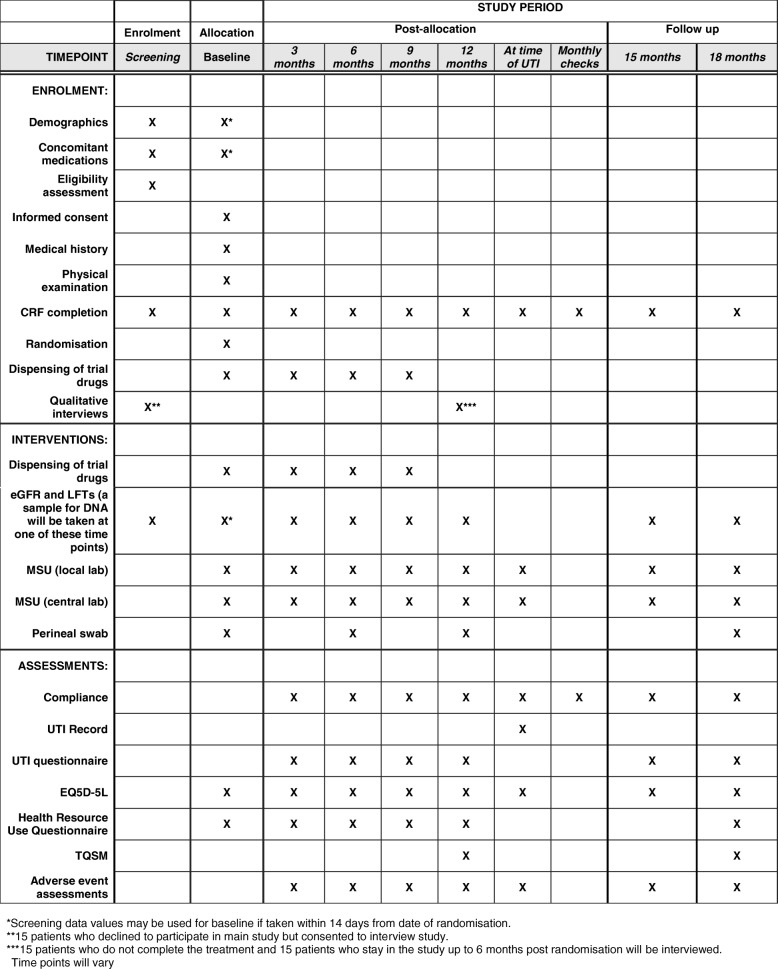
Standard Protocol Items: Recommendations for Interventional Trials (SPIRIT) Figure for the ALTAR trial

This protocol has been written in accordance with the Standard Protocol Items: Recommendations for Interventional Trials (SPIRIT) guidelines. Please refer to the SPIRIT Checklist and Figure (Fig. 2) submitted alongside this publication for further details (see Additional file 1).

## Discussion

As a pragmatically designed trial, apart from randomisation to one of the treatment options and participant completion of diaries and questionnaires; participants will follow standard care pathways in NHS secondary care. Both prophylactic antibiotics and methenamine hippurate are licensed and approved for routine NHS use against rUTI and are standard care for this indication. Therefore, is it considered that the risk associated with trial participation is no higher than that of standard care. On this basis the trial was submitted to MHRA and given authorisation as a ‘Type A’, low-risk, notification-only study. A research study providing robust evidence of at least no-worse effectiveness for non-antibiotic treatment is needed to inform choices of alternative treatments to prolonged antibiotic use. This study aims to provide this in the context of a routine NHS care setting and it is hoped the pragmatic nature of the trial will ensure the generalisability of the results to the patient group that is the focus of this study.

### Trial status

Recruitment was ongoing at the point of submission. Since submission, the ALTAR trial has recruited to target ahead of expected time.

## Additional file

Additional file 1: Standard Protocol Items: Recommendations for Interventional Trials (SPIRIT) 2013 Checklist: recommended items to address in a clinical trial protocol and related documents. (DOC 119 kb)

## Abbreviations

ABU: Asymptomatic bacteriuria
AE: Adverse event
AR: Adverse reaction
CA: Competent authority
CDI: *Clostridium difficile* infection
CI: Chief investigator
CRF: Case Report Form
DMC: Data Monitoring Committee
*E. coli*: *Escherichia coli*
eCRF: Electronic Case Report Form
eGFR: Estimated glomerular filtration rate
EQ5D-5 L: EuroQoL 5 Dimension Questionnaire
GCP: Good Clinical Practice
HTA: Health Technology Assessment
IMP: Investigational medicinal product
ISF: Investigator Site File
ISRCTN: International Standard Randomised Controlled Trials Number
LCRN: Local Clinical Research Network
LFT: Liver function tests
MHRA: Medicines and Healthcare products Regulatory Agency
MRSA: Methicillin-resistant *Staphylococcus aureus*
MSU: Mid-stream urine
NCTU: Newcastle Clinical Trials Unit
NHS: National Health Service
PHE: Public Health England
PI: Principal investigator
PIC: Participant identification centre
rUTI: Recurrent urinary tract infection
SAE: Serious adverse event
SAR: Serious adverse reaction
SDV: Source Data Verification
TMF: Trial Master File
TMG: Trial Management Group
TQSM: Treatment Questionnaire on Satisfaction with Medication
TSC: Trial Steering Committee
UTI: Urinary tract infection

## References

[1] Laupland KB, Ross T, Pitout JD (2007). Community-onset urinary tract infections: a population based assessment. Infection.

[2] Ikaheimo R, Sutonen A, Heiskanen T, Karkkainen U, Kuosmanen P (1996). Recurrence of urinary tract infection in a primary care setting: analysis of a 1-year follow-up of 179 women. Clin Infect Dis.

[3] 2011 Census. Population Estimates for the United Kingdom: Office for National Statistics; 2011. Retrieved September 2013 https://www.ons.gov.uk/peoplepopulationandcommunity/populationandmigration/populationestimates/bulletins/2011censuspopulationestimatesfortheunitedkingdom/2012-12-17.

[4] Albert X, Huertas I, Pereiro I, Sanfélix J, Gosalbes V, Perrotta C. Antibiotics for preventing recurrent urinary tract infection in non-pregnant women. Cochrane Database Syst Rev 2004, Issue 3. Art. No.: CD001209. doi: 10.1002/14651858.CD001209.pub2. 10.1002/14651858.CD001209.pub2 PMC7032641 15266443

[5] Beerepoot MA, Ter Riet G, Nys S, van der Wal WM, de Borgie CA (2012). Lactobacilli vs antibiotics to prevent urinary tract infections: a randomized, double-blind, non-inferiority trial in postmenopausal women. Arch Intern Med.

[6] Department of Health (2000). UK antimicrobial resistance strategy and action plan.

[7] Healthcare Associated Infection Task Force. The Scottish management of antimicrobial resistance action plan [ScotMARAP] 2008. Edinburgh: Scottish Government. p. 2008.

[8] Scottish Intercollegiate Guidelines Network (SIGN). Management of suspected bacterial urinary tract infection in adults. Edinburgh: SIGN; 2012. (SIGN publication no. 88). [July 2012]. Available from URL: http://www.sign.ac.uk. Accessed 1 Nov 2018.

[9] Urological Infections. 2013 European Association of Urology Guidelines. ISBN 978-90-79754-71-7.

[10] Lee BSB, Bhuta T, Simpson JM, Craig JC. Methenamine hippurate for preventing urinary tract infections. Cochrane Database Syst Rev 2012, Issue 10. Art. No.: CD003265. doi:10.1002/14651858.CD003265.pub3 10.1002/14651858.CD003265 11869659

[11] Murray BE, Rensimer ER, DuPont HL (1982). Emergence of high-level trimethoprim resistance in fecal *Escherichia coli* during oral administration of trimethoprim or trimethoprim–sulfamethoxazole. N Engl J Med.

[12] Kahlmeter G, Menday P (2003). Cross-resistance and associated resistance in 2478 *Escherichia coli* isolates from the Pan-European ECO.SENS Project surveying the antimicrobial susceptibility of pathogens from uncomplicated urinary tract infections. J Antimicrob Chemother.

[13] UK antmicrobial resistance strategy. Department of Health. London; 2013. www.gov.uk/dh. Accessed 5 Oct 2018.

[14] Gupta K, Hooton TM, Naber KG, Infectious Diseases Society of America; European Society for Microbiology and Infectious Diseases (2011). International clinical practice guidelines for the treatment of acute uncomplicated cystitis and pyelonephritis in women: a 2010 update by the Infectious Diseases Society of America and the European Society for Microbiology and Infectious Diseases. Clin InfectDis.

[15] Kumarasamy KK, Toleman MA, Walsh TR (2010). Emergence of a new antibiotic resistance mechanism in India, Pakistan, and the UK: a molecular, biological, and epidemiological study. Lancet Infect Dis.

[16] Scottish Medicines Consortium, Scottish Antimicrobial Prescribing Group (2012). Good practice recommendations for hospital antimicrobial stewardship in NHS Scotland.

[17] Health Protection Scotland (2012). Quarterly report on the surveillance of Clostridium difficile infection (CDI) in Scotland, October–December 2011.

[18] MAJ B, Geerlings SE, van Haarst EP, Mensing van Charante N, Riet G (2013). Nonantibiotic prophylaxis for recurrent urinary tract infections: a systematic review and meta-analysis of randomized controlled trials. J Urol.

[19] Atkinson MJ, Sinha A, Hass SL, Colman SS, Kumar RN, Brod M (2004). Validation of a general measure of treatment satisfaction, the Treatment Satisfaction Questionnaire for Medication (TSQM), using a national panel study of chronic disease. Health Qual Life Outcomes.

[20] Curtis L (2007). Unit costs of health and social care.

[21] Drummond M, Sculpher M, Torrance G, O'Brien B, Stoddart G (2005). Methods for the economic evaluation of health care programmes.

[22] Philips Z, Ginnelly L, Sculpher M, Claxton K, Golder S, Riemsma R (2004). Review of guidelines for good practice in decision-analytic modelling in health technology assessment. Health Technol Assess.

[23] Guide to the methods of technology appraisal. London: National Institute for Health and Clinical Excellence, 2008; [accessed July 2015]. URL: https://www.nice.org.uk/process/pmg9/chapter/foreword.

[24] Caro JJ, Briggs AH, Siebert U (2012). Modelling good research practices—overview: a report of the ISPOR-SMDM modeling good research practices task force-1. Value Health.

[25] Devlin N, Shah K, Feng Y, Mulhern B, van Hout B (2018). Valuing health-related quality of life: an EQ-5D-5L value set for England. Health Econ.

[26] Public Health England. UK Standards for Microbiology Investigations (UK SMI) B 41: Investigation of Urine. 2018. URL: https://www.gov.uk/government/publications/smi-b-41-investigation-of-urine. Accessed 5 Oct 2018.

[27] The European Committee on Antimicrobial Susceptibility Testing (2017). Breakpoint tables for interpretation of MICs and zone diameters, version 7.1.

